# Rhizosphere effects and microbial N limitations drive the root N limitations in the rhizosphere during secondary succession in a *Pinus tabuliformis* forest in North China

**DOI:** 10.3389/fpls.2024.1392934

**Published:** 2024-07-30

**Authors:** Songlan Duan, Jinping Guo, Yunxiang Zhang, Libao Liu, Rui Wang, Rongrong Zheng

**Affiliations:** College of Forestry, Shanxi Agricultural University, Jinzhong, China

**Keywords:** rhizosphere effects, N:P stoichiometry, N limitation, stand ages, *Pinus tabuliformis*

## Abstract

**Introduction:**

Rhizosphere effects (REs) have recently been identified as important regulators of root and microbial nutrient acquisition and are positively involved in nutrient cycling of belowground carbon (C), nitrogen (N), and phosphorus (P). Nutrient conditions of the fine roots and soil N are likely to influence REs. Still, it is unclear how REs of soil nutrients themselves variably impact the supply of nutrients to plants in terms of the responses to soil N due to succession.

**Methods:**

In this study, we applied both fine roots and extracellular enzymes for vector analysis and stoichiometry of N:P to explore the metabolic limitations of roots and rhizospheric soil microbes and their relationships with REs across five levels of soil N (0, 5, 10, 15, and 20 kg N m^−2^ year^−1^) along successional age classes of 42, 55, and 65 years in a *Pinus tabuliformis* forest.

**Results:**

Overall, the metabolism of root and rhizospheric soil microbes was mediated by soil N. N limitation of roots initially decreased before increasing, whereas that of microbes demonstrated opposite trends to the N levels owing to competition for inorganic N between them by REs of NO_3_^−^–N. However, N limitations of both roots and microbes were alleviated in young stands and increased with succession after the application of N. In addition, root N limitations were manipulated by REs of three different soil N-related indicators, i.e., total N, NH_4_^+^–N, and NO_3_^−^–N. Rhizospheric soil microbial N limitation was almost unaffected by REs due to their strong homeostasis but was an important driver in the regulation of root N limitation.

**Discussion:**

Our results indicated that successional age was the most critical driver that directly and indirectly affected root N metabolism. However, the level of N application had a slight effect on root N limitation. Microbial N limitation and variations in the REs of N indicators regulated root N limitation in the rhizosphere. As a result, roots utilized REs to sequester N to alleviate N limitations. These findings contribute to novel mechanistic perspectives on the sustainability of N nutrition by regulating N cycling in a system of plant–soil–microbes in the rhizosphere to adapt to global N deposition or the heterogeneous distribution of bioavailable soil N with succession.

## 1 Introduction

Over the last decade, there has been a rapid increase in the investigation of the rhizosphere, which is strongly affected by roots and serves as an important area of research on soil–plant–microbial interactions (Philippot et al., 2013; Vives-Peris et al., 2020; Ling et al., 2022; Jing et al., 2023; López et al., 2023) because of its critical role in terrestrial carbon (C) and nitrogen (N) cycling (Cheng et al., 2014; Finzi et al., 2015; Gan et al., 2021). Decoding the course of the rhizosphere connected with N cycling and their responses to exogenous N input has significant effects on plant physiological metabolism and growth in different life stages (Kuzyakov and Xu, 2013; Kang et al., 2022). The release of a diverse array of chemicals, including carbohydrates, organic acids, amino acids, mucilage, and exudative root cells, into the rhizosphere by living roots during plant growth and metabolism favors the improvement of soil fertility (Cheng and Kuzyakov, 2015; Carrillo et al., 2017; Vives-Peris et al., 2020). For example, *Zea mays* release more carbohydrates and c-aminobutyric acid into the rhizosphere to modify the P deficiency while decreasing the excretion of amino acids and the quantities of sugars to combat N and potassium (K) deficiencies (Carvalhais et al., 2011; Olanrewaju et al., 2019; Koshila Ravi and Muthukumar, 2024). This process leads to significant differences between rhizospheric soils that cling to root surfaces and bulk soils in terms of their physical, chemical, and biological properties (Kumar and Garkoti, 2022; Li et al., 2022; Herre et al., 2022a, 2022b). Specifically, it promotes the growth and development of plants, carbon sequestration, and functioning of terrestrial ecosystems by enhancing soil nutrient availability, influencing plant hormonal signaling, facilitating nutrient absorption and cycling, and alleviating abiotic stress through microorganisms in the rhizosphere (Kumar and Verma, 2019; Berger et al., 2020; Darriaut et al., 2022; Solomon et al., 2024; Zhuang et al., 2024). The magnitude of these modifications to rhizosphere attributes is often defined as the rhizosphere effect (RE) (Phillips and Fahey, 2006; Finzi et al., 2015; Gan et al., 2021).

The ecological stoichiometry of the N:P ratio, an effective indicator for detecting nutritional limitation, describes the interactions of essential elements in the global ecological processes of the balance between energy flow and nutrient cycles in terrestrial ecosystems (Elser et al., 2010; Chen et al., 2016; Ren et al., 2016; Cui et al., 2018). Nitrogen and phosphorus, two basic but the most restrictive nutritional elements, directly affect the growth and development of plants, the nutrient status of the soil, and the metabolism and activities of microbes (Elser et al., 2000, 2010; Sinsabaugh and Follstad Shah, 2011, 2012; Hu et al., 2018). Roots are essential organs that facilitate the absorption of N, P, and other elemental nutrients and serve as an interface between the soil and plants (Wang et al., 2020; Shi et al., 2021). The N:P ratios in fine roots are the most immediate and efficient reflections of growth rate and physiological adjustment to the environment, such as changes in N levels, warming, and elevated CO_2_ (Sardans et al., 2017; Wang et al., 2020), as well as nutrient availability and limitations (Su et al., 2019; Cao et al., 2020; Geng and Jin, 2022; Ma et al., 2022; Geng et al., 2023). Furthermore, the ecological stoichiometry of extracellular enzyme stoichiometry (EES) actively engaged in N and P cycling revealed equilibrium relationships between the relevant nutrient requirements for microbial metabolism and soil provision (Sinsabaugh et al., 2009; Sinsabaugh and Follstad Shah, 2012; Cui et al., 2019; Dong et al., 2021). Subsequently, the proposal and development of the threshold elemental ratio (TER) and enzymatic vector (V-T) models have thoroughly estimated the metabolic limitations of microbes based on traditional models, enhanced the uniformity of predictions for the metabolic limitations of microbes, and further confined the nutritional constraints of N and P (Sinsabaugh et al., 2009; Sinsabaugh and Follstad Shah, 2012; Cui et al., 2018, 2021).

Microbes use their ability to decompose soil organic matter (SOM) to acquire supplemental nutrients such as N and P via labile C as root exudates released by plant roots (Phillips et al., 2011; Dijkstra et al., 2021; He et al., 2021). REs are influenced by the availability of soil nutrients owing to the metabolic balance between microbes and fine roots (Phillips and Fahey, 2008) and may also be a useful nutritional adjunct to plants (Dijkstra et al., 2013; Hicks et al., 2020), which further affects soil C and N cycling (Dijkstra et al., 2013; He et al., 2021). REs also contribute to the maintenance of forest productivity during extra growth and provide a long-term enhancement response to elevated CO_2_ when forests endure a gradual increase in N limitation (Drake et al., 2011; Phillips et al., 2011; Meier et al., 2015). For example, a meta-analysis reported that roots could accelerate mineralization, and the priming of REs explained approximately 33.3% of the total soil C and N mineralization. Furthermore, the REs due to root-derived C accounted for up to 4% and 6% of gross and net primary production, respectively, in a temperate forest (Finzi et al., 2015). Therefore, the magnitude of REs is not only a crucial factor used to explain species coexistence and biodiversity (Lambers et al., 2009; Cheng et al., 2014; Hicks et al., 2020) but also a promising mechanism for evaluating the capacity of plants to accelerate soil organic carbon (SOC) turnover/accumulation (Han et al., 2020; Dijkstra et al., 2021) and resist future atmospheric N deposition (Phillips et al., 2011; Henneron et al., 2020; Gao et al., 2023). REs are emerging as a global nutritional acquisition strategy for different types of woody plants at large spatial scales and function as substitutional nutrients when bulk soil nutrients are insufficient for plant growth (Dijkstra et al., 2013; Sun et al., 2023).

Heterogeneity in the distribution of bioavailable N in soil across different regions and the incessant increase in N deposition in the atmosphere (Sun et al., 2022; McDonnell et al., 2023; Zhou et al., 2024) have led to differences in the nutrient limitation of roots and rhizospheric soil microbes, as well as rhizospheric effects that can extend through the entire ecosystem (Phillips et al., 2011; Zhang et al., 2019; Cai et al., 2023). Several studies have focused on regional and global climatic change (Dijkstra et al., 2013; Cheng et al., 2014; Terrer et al., 2016) and plant economic resource acquisition strategies (Keller et al., 2021; Sun et al., 2023). Although N deposition is a major global change, the response of REs to changes in soil N content remains poorly understood. Thus, an investigation into the effects of N application on the REs of soil nutrients linked to N and P and root and rhizospheric soil microbial nutrient limitations is critical. Specifically, it is critical to elucidate the ecological influences of N deposition on the nutritional balance and constraints in the rhizosphere of forest ecosystems.

*Pinus tabuliformis* is a widespread ectomycorrhizal (ECM) tree that occupies a pioneering niche in the coniferous forest ecosystems of northern China (Long et al., 2016; Taniguchi et al., 2021) and plays a dominant role as a carbon sink in forests (Song et al., 2021). This native tree species is drought-resistant and adaptable to harsh environments; therefore, it is typically used for revegetation (Song and Hou, 2020; Zhang et al., 2021). With continuous increases in N deposition in the atmosphere and the heterogeneity of the distribution of bioavailable N in the soil across different regions, it is essential to understand the responses of REs, the ecological stoichiometry of the C:N:P ratio, and the nutritional limitations of roots and microbes in the succession of *P. tabuliformis* forests. In this study, the metabolic limitations of both fine roots and rhizospheric soil microbes were explored by means of vector analysis of extracellular enzymes and roots based on the stoichiometry of N:P and their relationships with REs across five levels of N application (0, 5, 10, 15, and 20 kg N m^−2^ year^−1^) along a secondary succession of classes (42, 55, and 65) in a *P. tabuliformis* forest. Specifically, we hypothesized that 1) the application of N affects the nutritional limitations of rhizospheric soil microorganisms and fine roots, as well as REs. Furthermore, the response of nutritional microbial limitations in rhizospheric soil and roots of elder stands was more susceptible than that of young stands. 2) The nutritional limitation of roots with succession would be associated with microbial nutritional limitation in the rhizosphere and the REs.

## 2 Methods

### 2.1 Site description

**Figure 1:**
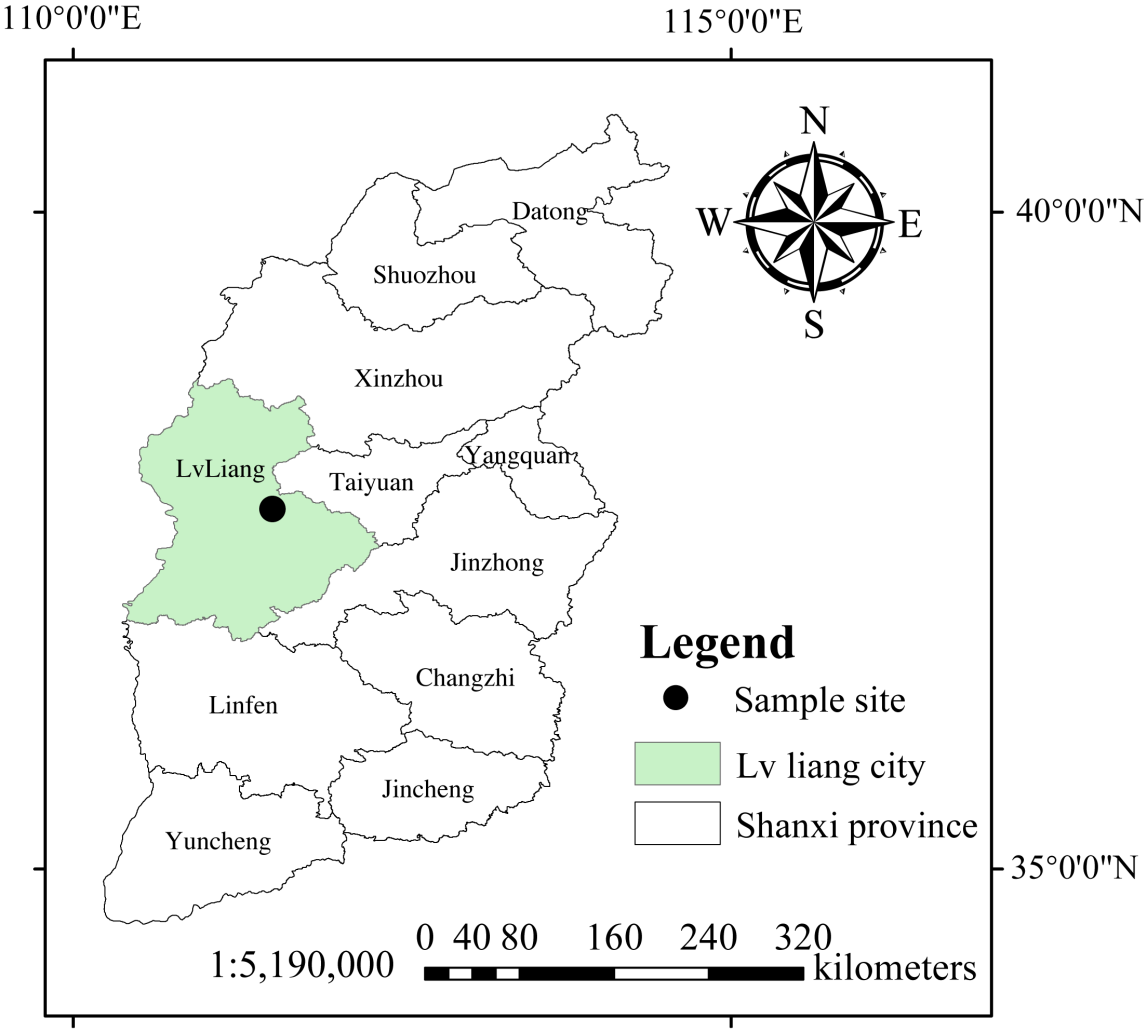
Location of the sample site.

This study was conducted at the Xiaowenshan Forest Farm (established in 1962) in the Guandishan Forest Region of Shanxi Province, northern China (**Figure 1**). The total area covers approximately 1.99 × 10^4^ ha, with altitudes ranging from 1,460 to 1,610 m. This region is located in the inner continental mountain monsoon climate zone. The mean annual temperature is 4.2°C, the mean annual precipitation is 822.6 mm, and the relative humidity is 70.9%. The soil is a typical Alfisol, which is covered by a 3–7-cm humus layer. *P. tabuliformis* is a typical dominant tree species, with a few associated species such as *Larix principis-rupprechtii*, *Picea wilsonii*, *Betula platyphylla*, and *Quercus mongolica*. The understory species primarily comprise *Spiraea salicifolia*, *Rosa xanthine*, *Corylus mandshurica*, *Rosa bella*, and a few *Lespedeza bicolor* (Zhang et al., 2021).

### 2.2 Experimental design

In 2020, 42-year-old (42-year), 55-year-old (55-year), and 65-year-old (65-year) stands of *P. tabuliformis* forest were selected as experimental sites at the forest farm (111°24′E–112°37′E, 37°41′N–37°54′N) (**Figure 1**). A randomized block design with three replicate blocks of five levels of N application at each successional age was established before the experiment: N_0_ (0 kg N m^−2^ year^−1^, N_1_ (5 kg N m^−2^ year^−1^), N_2_ (10 kg N m^−2^ year^−1^), N_3_ (15 kg N m^−2^ year^−1^), and N_4_ (20 kg N m^−2^ year^−1^). Five 201-m^2^ circular plots (radius = 8 m) with a buffer zone (Song, 2017; Pretzsch, 2022) were included in each block. A backpack sprayer was used to apply various concentrations of urea solutions (CH_4_N_2_O) to the corresponding N application plots (N_0_, 0 g CH_4_N_2_O; N_1_, 6,608 g CH_4_N_2_O; N_2_, 8,913 g CH_4_N_2_O; N_3_, 11,217 g CH_4_N_2_O; and N_4_, 15,826 g CH_4_N_2_O; each added to 20 L of water) continuously before rain in mid-May, July, and September of each year (Geng and Jin, 2022). The details of the experimental sites are listed in **Table 1**.

**Table 1:** Basic data on the sampling points in the study area.

Stand age	Block	Altitude (m)	Longitude	Latitude	Slope (°)	Mean diameter at breast height (DBH) (cm)	Mean tree height (m)	Canopy closure (%)
42 years	B1	1495.2	111°30′43.83″E	37°44′08.75″N	19°	24.03	16.97	75
	B2	1539.8	111°31′29.14″E	37°44′12.27″N	22°	27.28	16.24	52
	B3	1554.4	111°31′30.40″E	37°44′14.57″N	21°	27.73	16.64	59
55 years	B1	1523.8	111°30′48.03″E	37°44′15.90″N	32°	23.18	14.45	42
	B2	1554.3	111°30′53.64″E	37°44′24.39″N	35°	32.01	19.14	75
	B3	1566.2	111°30′56.99″E	37°44′25.69″N	37°	20.58	16.91	62
65 years	B1	1518.2	111°30′46.79″E	37°44′12.87″N	26°	26.39	16.76	80
	B2	1562.6	111°30′51.13″E	37°44′20.55″N	31°	29.99	16.75	60
	B3	1557.0	111°30′55.05″E	37°44′23.82″N	28°	24.07	18.47	67

### 2.3 Soil and root sampling

In June 2022, after the continuous application of N for 1.5 years, three standard trees were randomly selected to collect the paired non-rhizospheric and rhizospheric soils, as well as the living fine roots (Ø ≤ 2 mm) in each plot of each stand age. Three 8 cm inside diameter soil cores (10 cm depth) were randomly extracted under the canopy area of each standard tree to ensure sufficient rhizospheric soil and living fine root samples following careful removal of understory plants and surface litter. The living roots of *P. tabuliformis* can be distinguished based on their features, including shape, color, taste, and elasticity. The fine roots were gently shaken to collect the adhered rhizospheric soil (Phillips et al., 2011). The fine roots were collected and placed in polyethylene bags. Finally, soil without any attachment to the roots was collected, which was regarded as non-rhizospheric soil. The living fine roots and paired non-rhizospheric and rhizospheric soils of three standard trees collected from each plot were pooled for a homogenized sample.

A cooler box was used to store the collected rhizospheric and non-rhizospheric soil samples, and it was ensured that their transport to the laboratory was complete within 24 h of analysis. Each soil sample was divided into two fractions. One fraction was stored at 4°C for <1 week and subsequently used for the analysis of soil enzyme activity. The other fraction was sieved using a 0.25-mm sieve after air-drying at room temperature (20°C–25°C) to analyze its chemical properties, including the concentration of SOC, total N (TN), total P (TP), NH_4_^+^–N, NO_3_^−^–N, and available P (AP). The root samples were first gently rinsed in distilled deionized water over a 0.5-mm wet sieve and then dried to a constant mass at 75°C for 48 h after heat-killing at 105°C for 15 min. Finally, the dried root samples were ground into fine powder to determine their chemical properties [TN, TP, and total C (TC)].

### 2.4 Lab analysis of soil and root samples

The SOC and TC of the roots were quantified using a TOC/TN analyzer (Multi N/C 3100; Analytik Jena, Jena, Germany). The TN and TP concentrations in the paired rhizospheric and non-rhizospheric soils were quantified using Kjeldahl digestion and molybdenum blue colorimetry, respectively. Root TN and TP contents were determined by initial digestion with H_2_SO_4_–H_2_O_2_ (Nelson and Sommers, 1973; Parkinson and Allen, 1975). The soil samples were extracted using a 2 M KCl and 0.5 M NaHCO_3_ solution under shaking and then passed through filter paper to determine the available N and P (NH_4_^+^–N, NO_3_^−^–N, and AP) of paired rhizospheric and non-rhizospheric soils (Olsen et al., 1954; Bremner and Keeney, 1966). These parameters were determined using an automated discrete analyzer (SmartChem 450, AMS Alliance, Rome, Italy).

The activities of four soil extracellular enzymes, i.e., β-1,4-glucosidase (BG), β-1,4-*N*-acetylglucosaminidase (NAG), l-leucine aminopeptidase (LAP), and acid phosphatase (ACP), representing one C-acquiring, two N-acquiring, and one P-acquiring enzymes, respectively, were determined using standard fluorometric techniques and enzyme calibration (M Plex, Tecan, Männedorf, Switzerland) (Cui et al., 2019, 2021; Huang et al., 2022). The enzymatic activity was expressed as nmol g^−1^ h^−1^.

### 2.5 Data analysis

The REs of each soil nutrient variable were calculated as the percentage difference between paired rhizospheric and non-rhizospheric soil samples using the Equation 1 (Phillips and Fahey, 2006):


(1) $$\text{REs}=\left({\text{C}}_{\text{R}}-{\text{C}}_{\text{N}}\right)/{\text{C}}_{\text{N}}\times 100$$

where C_R_ and C_N_ are the concentrations of the measured variables that include one soil C-related indicator (SOC), three soil N-related indicators (TN, NH_4_^+^–N, and NO_3_^−^–N), and two soil P-related indicators (TP and AP) in the paired rhizospheric and non-rhizospheric soil samples, respectively.

Extracellular enzyme N:P stoichiometry in the rhizospheric soil was calculated using the Equation 2:


(2) $$\text{N}:{\text{P}}_{\text{SEE}}=Ln\left(NAG+LAP\right)/Ln\left(ACP\right)$$

where N:P_SEE_ represents the natural logarithm of the ratio of relative enzymatic activity, which indicates N acquisition and P acquisition in rhizospheric soil (Cui et al., 2018).

The vector analysis of soil extracellular enzymes was widely applied to determine the responses of soil microbial nutritional constraints of C, N, and P to internal plant succession and external soil environmental changes, which are always calculated with Equations 3 and 4 (Cui et al., 2021; Huang et al., 2022; Kang et al., 2022; Xu et al., 2022):


(3) $$\text{Vector length}=\sqrt[{2}]{{\left[\frac{Ln\left(BG\right)}{Ln\left(ACP\right)}\right]}^{2}+{\left[\frac{Ln\left(BG\right)}{Ln\left(NAG\;+\;LAP\right)}\right]}^{2}}$$

(4) $$\text{Vector angle}=Degree\left\{ATAN2\left[\left(\frac{Ln\left(BG\right)}{Ln\left(ACP\right)}\right),\left(\frac{Ln\left(BG\right)}{Ln\left(NAG\;+\;LAP\right)}\right)\right]\right\}$$

The vector length (dimensionless) quantified the relative microbial C limitation. The greater the vector length, the more severe the C limitation experienced by the microbes. The magnitude of the vector angle (°) determines the relative microbial N or P limitation. Vector angles (> 45° or < 45°) indicate that the microbes experienced relative P or N limitation, respectively. The greater the vector angle, the more P-limited the microbes and *vice versa* for N-limited microbes. Thus, we developed a new indicator to define microbial N limitation, as the formula, Equation 5:


(5) $$\text{Microbial N limitation}=Degree\left\{ATAN2\left[\left(\frac{Ln\left(BG\right)}{Ln\left(NAG\;+\;LAP\right)}\right),\left(\frac{Ln\left(BG\right)}{Ln\left(ACP\right)}\right)\right]\right\}$$

where *Degree* represents the tangent angle of the line extending from the origin of the coordinates to the points with horizontal and vertical coordinates of *Ln* BG/*Ln* ACP and *Ln* BG/*Ln* (NAG + LAP), respectively.

The N:P stoichiometry of fine roots was calculated as described by Elser, with Equation 6 (Elser et al., 2010):


(6) $$\text{N}:{\text{P}}_{\text{CON}}=TN/TP$$

where N:P_CON_ represents the mass ratio (g N/g P) of TN: TP in the fine roots.

A new vector analysis was developed to determine the nutritional limitations of N and P in plants, similar to that in a previous study (Xu et al., 2022), and was calculated with Equation 7:


(7) Vector angle=Degree[ATAN2(TP,TN)]

The magnitude of the vector angle (°) determines the relative N or P limitation of the fine roots. A vector angle (> 4.09° or < 3.58°) indicated that the roots were experiencing relative N or P limitation, respectively, and (3.58° < vector angle < 4.09°) indicated that the relative P or N was free from limitation in fine roots. The greater the vector angle, the more serious the N-limitation experience for the roots and *vice versa* for the P limitation. The degree represents the tangent angle of the line extending from the origin of the coordinates to points with the horizontal and vertical coordinates of the TN and TP contents, respectively.

Differences in the parameters representing the metabolic limitations of root and rhizospheric soil microbes and the REs between different N application levels and successional ages were tested using a two-way analysis of variance (ANOVA). Multiple comparisons among the different N application levels and successional ages were conducted using the least significant difference (LSD) test (*p* < 0.05). Relationships between paired nutrient REs were determined using Pearson’s correlation analysis. The relationships between root and rhizospheric soil microbial nutrient limitations and REs were identified using linear regression analysis.

The relative importance of TC content in fine roots, rhizospheric soil microbial nutrient limitation, and REs in the regulation of root nutrient limitation was demonstrated using random forest analysis and the random forest R package. The complicated directions of multiple variables and the direct and indirect effects of the predicted variables on the metabolic limitations of the roots were quantified by constructing structural equation models (SEMs) based on our fundamental knowledge in conjunction with the results of linear regression analysis and random forest modeling (Zhao et al., 2022). Moreover, we used five metrics containing low chi-square value (χ^2^), high p-values (> 0.05), high Jöreskog’s goodness fit index (GFI > 0.95), and comparative fit index (CFI > 0.95), as well as low root mean square error of approximation (RMSEA < 0.05) to verify an ideal model fit. Construction and examination of the SEM model were based on the maximum likelihood estimations and performed through the R “lavaan” package (Zhao et al., 2022). All analyses were performed using the R version 4.2.2 (R Core Team, 2022).

## 3 Results

### 3.1 Changes in root and rhizospheric soil microbial N limitations

#### 3.1.1 Effects of the application of N on root and rhizospheric soil microbial N limitations

**Figure 2:**
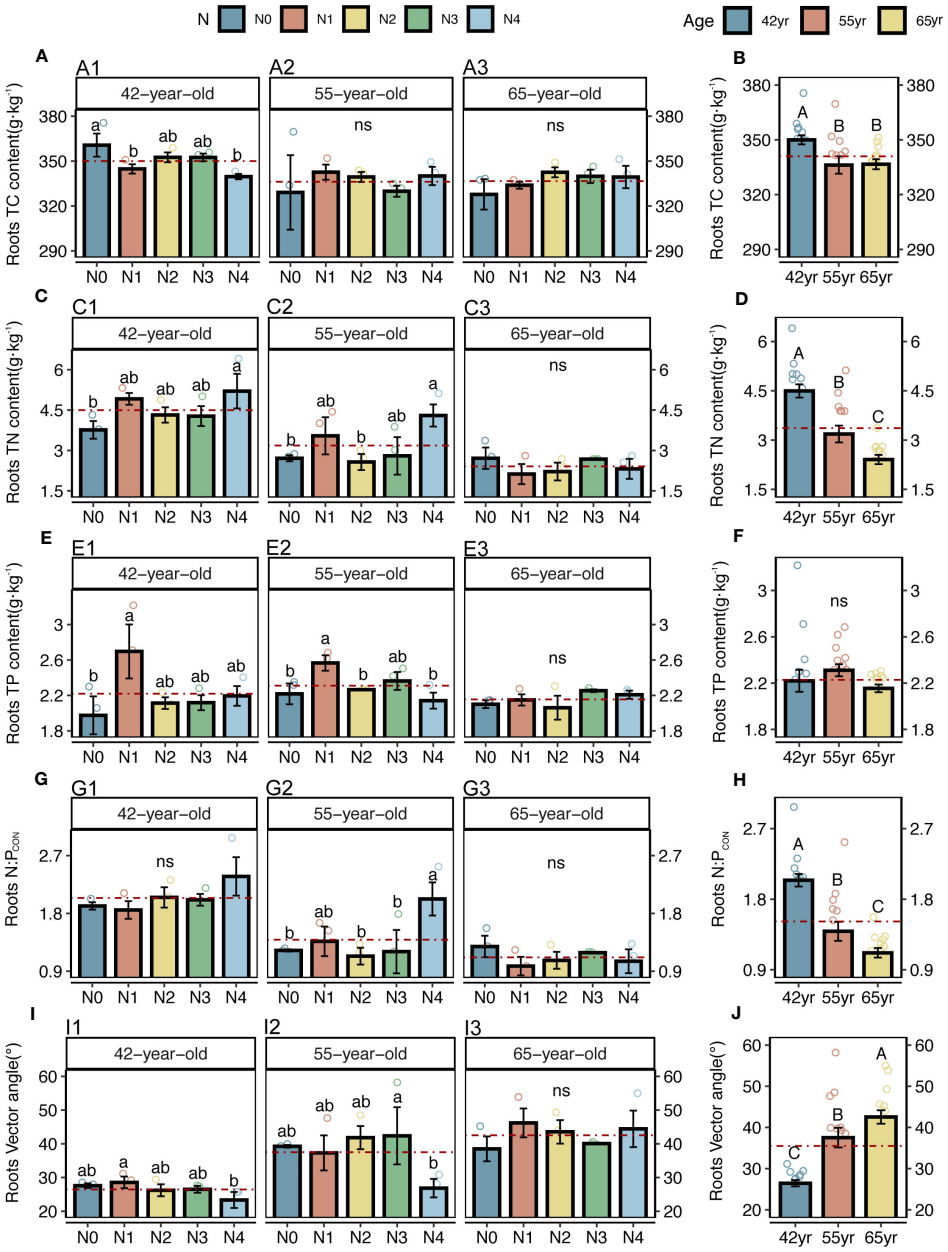
C, N, and P contents, N: P stoichiometry, and N limitations of fine roots for five levels of N application of stands of different ages **(A, C, E, G, I)** and their changes with succession **(B, D, F, H, J)** in *Pinus tabuliformis* forest. Different small letters denote the significant differences (*p* < 0.05) among different N application levels within the successional age, different capital letters denote the significant differences (*p* < 0.05) among three successional ages, and ns represents non-significance on the basis of ANOVA and LSD tests (*p* > 0.05). N0 = 0 kg N m^−2^ year^−1^; N1 = 5 kg N m^−2^ year^−1^; N2 = 10 kg N m^−2^ year^−1^; N3 = 15 kg N m^−2^ year^−1^; N4 = 20 kg N m^−2^ year^−1^. 42yr, 42-year-old stand; 55yr, 55-year-old stand; 65yr, 65-year-old stand. Red dashed lines represent the mean value of each variable in each successional age. LSD, least significant difference.

**Figure 3:**
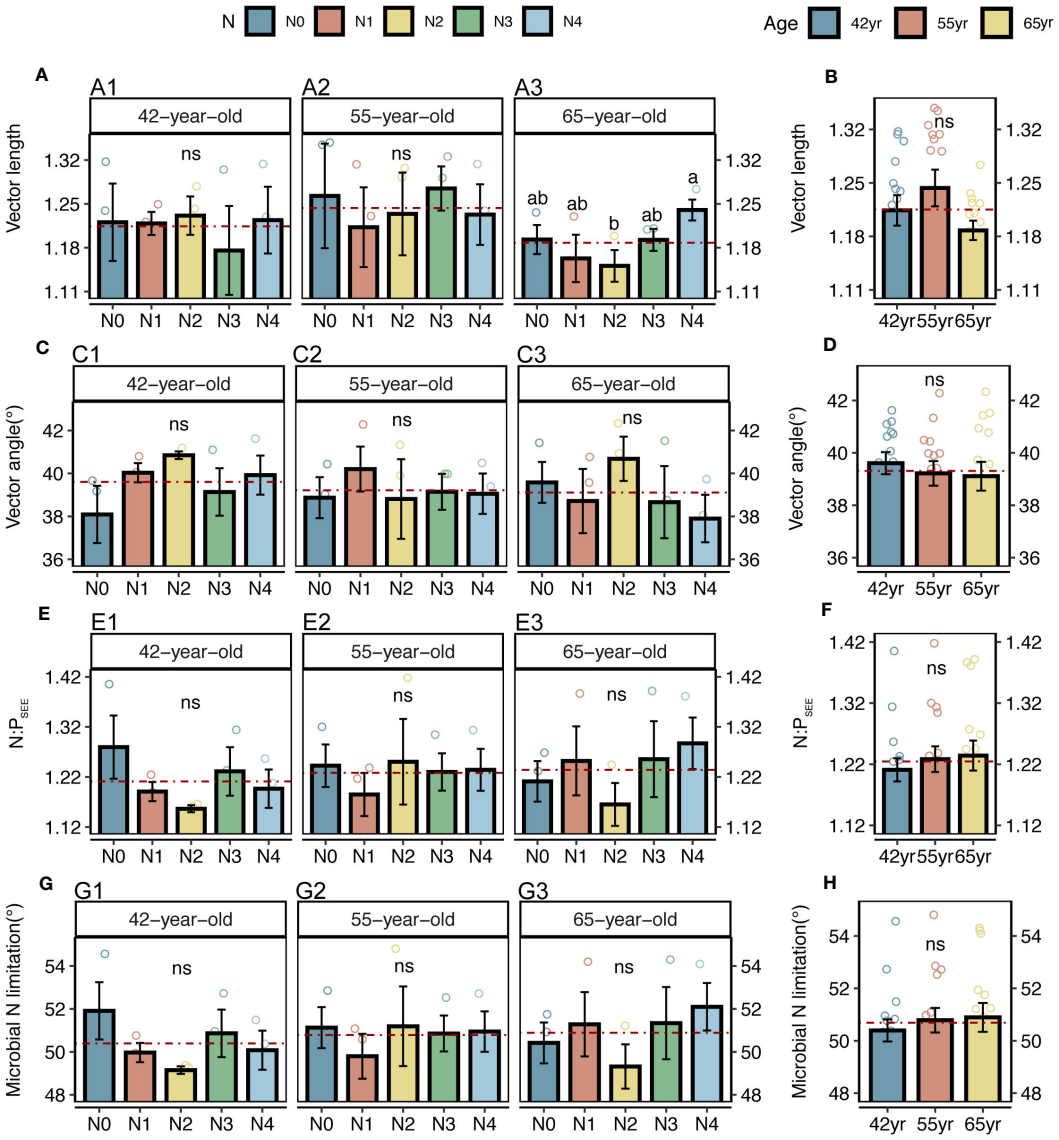
Vector analysis and stoichiometry N: P of extracellular enzyme, as well as N limitations of rhizospheric soil microbes for five levels of N application of stands of different ages **(A, C, E, G)** and their changes with succession **(B, D, F, H)** in *Pinus tabuliformis* forest. Different small letters denote the significant differences (*p* < 0.05) among different N application levels within the successional age, different capital letters denote the significant differences (*p* < 0.05) among three successional ages, and ns represents non-significance on the basis of ANOVA and LSD tests (*p* > 0.05). Red dashed lines represent the mean value of each variable in each successional age. LSD, least significant difference.

Both the roots and microbes in the rhizosphere exhibited serious N restrictions [root N:P_CON_ < 14, root vector angle (> 4.09°), enzyme N:P ratios > 1, and microbial vector angle (< 45°)] (**Figures 2G–J**, **3C–F**). Typically, the application of N generally increased the root TC, TN and TP contents of three successional ages (**Figures 2A, C, E**), but significantly decreased the root vector angles in both 42-year and 55-year stands (*p* < 0.05) (**Figure 2I**). This indicated an enhancement in nutrient uptake and a mitigation in root N limitation following the application of N. However, microbial N limitation in the rhizosphere of stands of three successional ages and the root N limitation in the 65-year stand were not affected by the application of N (*p* > 0.05) (**Figures 2I**, **3G**), although there was a slight decrease when the level of N application was >15 kg N m^−2^ year^−1^ in root N limitation of the 65-year stand. Vector length, representing a relative microbial C limitation, was only significantly different among the levels of N application in the 65-year stand (**Figure 3A**).

#### 3.1.2 Patterns of root and rhizospheric soil microbial N limitations with succession

**Figure 4:**
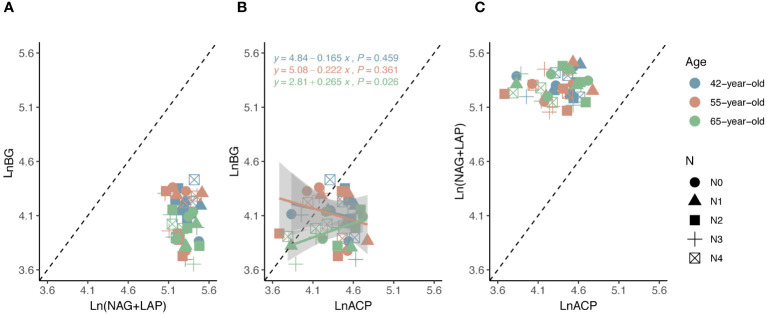
Standard major analysis of soil extracellular enzyme stoichiometry (C: N, C: P and N: P) **(A–C)** to identify the relative nutritional constraint for N or P of rhizospheric soil microbes. Black dashed lines denote referenced lines with slopes of 1.0 for the extracellular enzyme stoichiometry.

**Figure 5:**
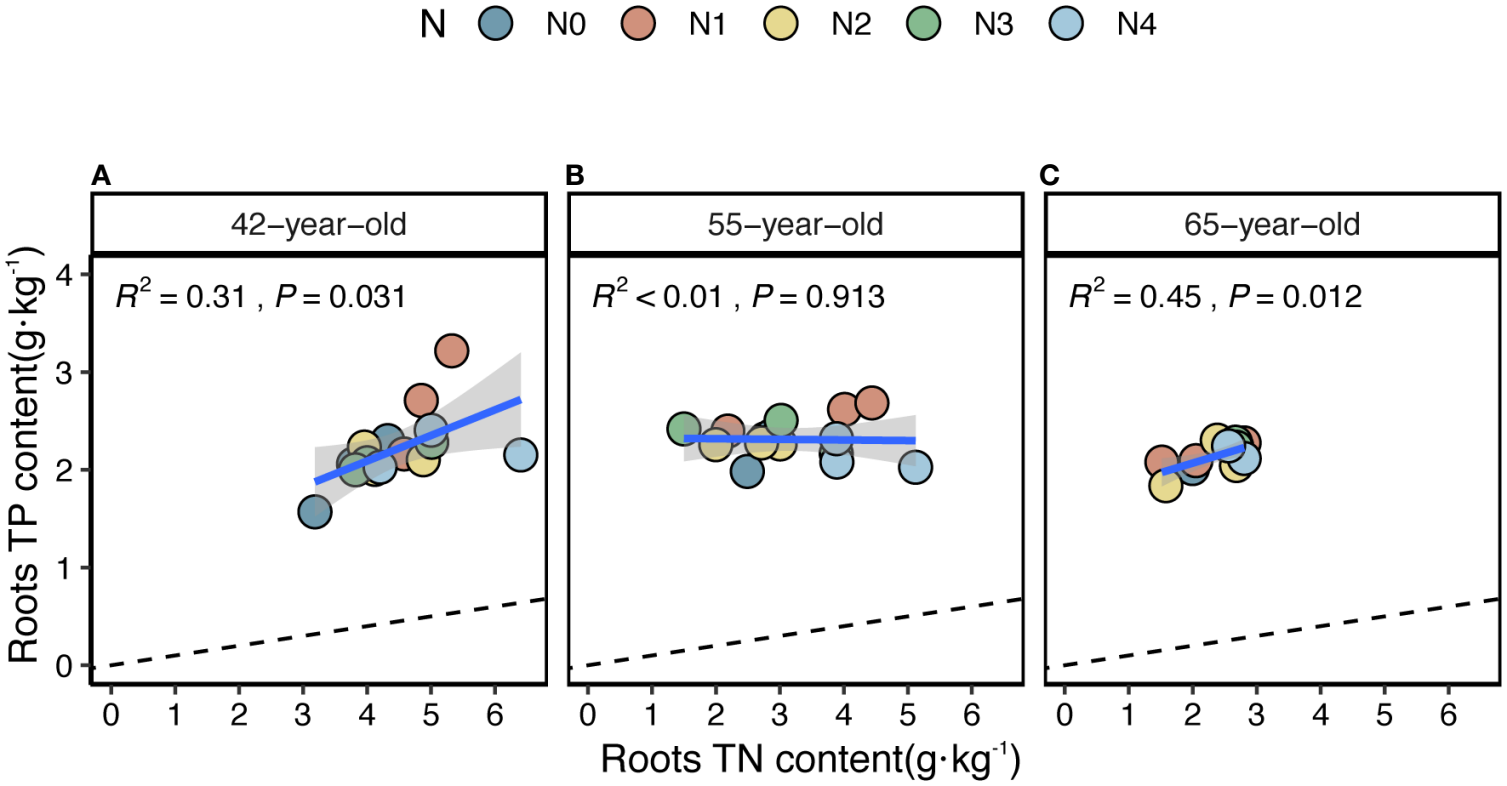
Simple linear regression analysis to identify the relative nutritional constraint for N or P of fine roots of stands of 42-, 55- **(A)**, **(B)** and 65-year-old **(C)**. Black dashed lines denote referenced lines with slopes of 14 for the N:P stoichiometry.

The average values of the natural logarithmic ratios of the extracellular enzyme activity represented stoichiometric C:N:P ratios of 1: 1.30: 1.08, 1: 1.28: 1.05, and 1: 1.34: 1.09 (**Figure 4**), demonstrating a slight increase with succession, although the effect was not significant (**Figures 3B, D, F, H**). However, succession had significant effects on root elements, except for the TP content, and significantly increased the root N limitation (**Figures 2B, D, F, H, J**). Besides, the root TN and TP contents were linearly correlated in all three stands (**Figure 5**). There were no interactive effects between the application of N and succession on root and rhizospheric soil microbial N limitations (*p* > 0.05; **Table 2**).

**Table 2:** Interaction effects of successional ages and the levels of N application tested by two-way ANOVA.

	Factor	F	*p*
Root properties	Root TC	0.937	0.5012
	Root TN	1.458	0.2141
	Root TP	1.527	0.18956
	Root N:P_CON_	1.495	0.201
	Root N limitation	1.336	0.264
Microbe properties	Vector length	0.364	0.931
	Vector angle	0.633	0.743
	N:P_SEE_	0.654	0.727
	Microbial N limitation	0.633	0.743
Rhizosphere effects	REs on SOC	1.195	0.3350
	REs on TN	0.840	0.575
	REs on TP	3.853	0.00318 **
	REs on NH_4_^+^–N	1.130	0.372
	REs on NO_3_^−^–N	0.494	0.851
	REs on AP	1.832	0.1099

TC, total C; TN, total N; TP, total P; SOC, soil organic carbon; REs, rhizosphere effects; AP, available P; **p < 0.01.

### 3.2 Changes in REs

#### 3.2.1 Effects of the application of N on REs

**Figure 6:**
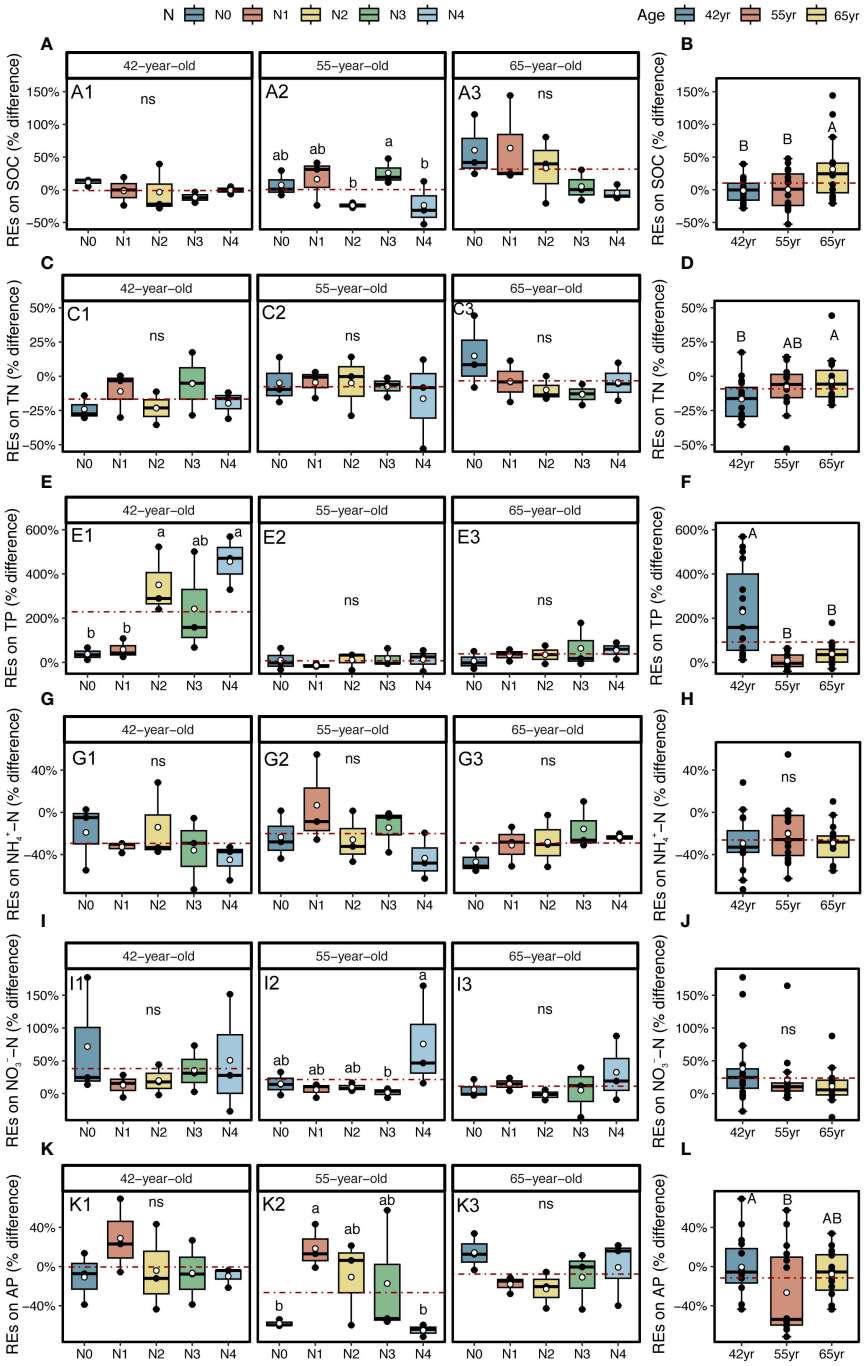
REs on SOC, TN, and TP, as well as available nutrients (NH_4_^+^–N, NO_3_^−^–N, and AP) for five levels of N application of stands of different ages **(A, C, E, G, I, K)** and their changes with succession **(B, D, F, H, J, L)** in *Pinus tabuliformis* forest. Different small letters denote the significant differences (*p* < 0.05) among different N application levels within the successional age, different capital letters denote the significant differences (*p* < 0.05) among three successional ages, and ns represents non-significance on the basis of ANOVA and LSD tests (*p* > 0.05). N0 = 0 kg N m^−2^ year^−1^; N1 = 5 kg N m^−2^ year^−1^; N2 = 10 kg N m^−2^ year^−1^; N3 = 15 kg N m^−2^ year^−1^; N4 = 20 kg N m^−2^ year^−1^. 42yr, 42-year-old stand; 55yr, 55-year-old stand; 65yr, 65-year-old stand. White points and red dashed lines represent the mean value of each variable at each N application level and in each successional age, respectively. REs, rhizosphere effects; SOC, soil organic carbon; TN, total N; TP, total P; AP, available P; LSD, least significant difference.

The application of N generally reduced the REs of SOC and TN in stands of three successional ages (except REs of TN in a 42-year stand), whereas it increased the REs of two soil P-related indicators, that is, TP and AP (except REs of AP in the 65-year stand) (**Figures 6A, C, E, K**). The REs of NH_4_^+^–N and NO_3_^−^–N demonstrated a similar pattern after the application of N, which decreased in the 42-year stand and increased in the 55-year and 65-year stands (**Figures 6G, I**).

**Figure 7:**
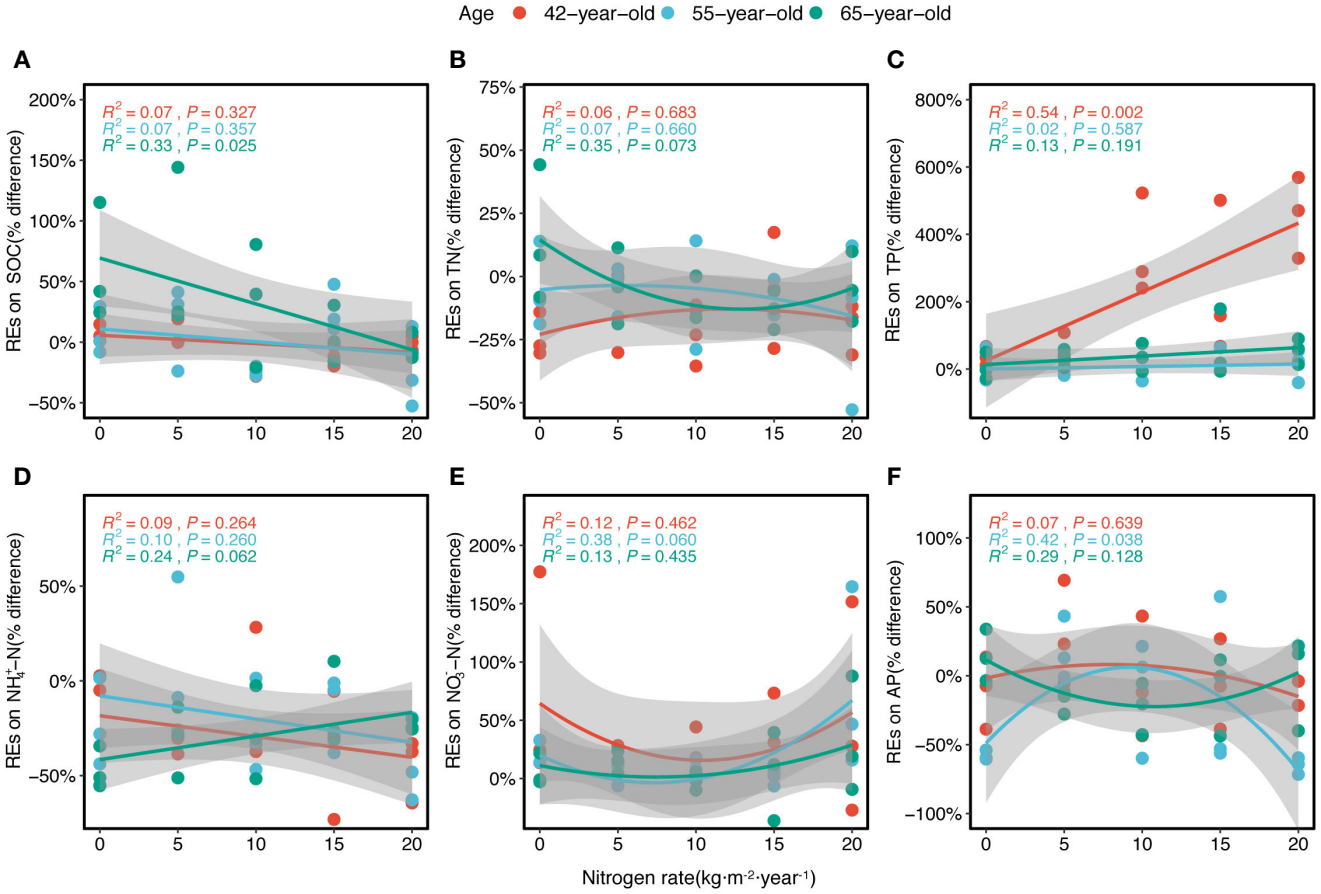
Regressions of the REs of six soil nutrients (SOC, TN, TP, NH_4_^+^–N, NO_3_^−^–N, and AP) along the levels of N application for three successional ages of *Pinus tabuliformis* forest **(A–F)**, respectively. Lines in three colors imply the fitted linear relationships. R^2^ and *P* represent the coefficient of determination and level of significance, respectively. Gray-shaded areas indicate the 95% confidence intervals. REs, rhizosphere effects; SOC, soil organic carbon; TN, total N; TP, total P; AP, available P.

The REs were generally significantly correlated with historical N rates according to the fitted model (**Figure 7**). The REs of the SOC and NH_4_^+^–N generally linearly decreased with N levels (except in the REs of NH_4_^+^–N in 65-year stand) (**Figures 7A, D**). The REs of TN and AP generally decreased non-linearly with increasing N levels and demonstrated a similar trend for both the 42-year and 55-year stands but differed from that of the 65-year stand (**Figures 7B, F**). However, the REs of NO_3_^−^–N generally increased non-linearly with higher N levels (**Figure 7E**). The REs of the TP increased linearly with the N level (**Figure 7C**), and the REs of the AP showed nearly no variation under different N levels (**Figure 6K**).

#### 3.2.2 Pattern of REs with succession

During the development of *P. tabuliformis* forests, REs differed following the application of N. The magnitudes of the REs of SOC and TN significantly increased with succession and were greater in the 65-year stands (**Figures 6B, D**). However, the REs of TP, NH_4_^+^–N, NO_3_^−^–N, and AP tended to decrease with succession and were only significant in REs of two soil P-related indicators, i.e., TP and AP (**Figures 6F, H, J, L**). The directions of TP and NO_3_^−^–N were positive and greater in magnitudes in 42-year stand compared with those of the other successional stages (**Figures 6F, J**). Generally, there were no interactive effects between N application and succession on REs, except for the REs of TP (*p* > 0.05; **Table 2**).

### 3.3 Relationships between the paired REs and root TC, TN, and TP contents

**Figure 8:**
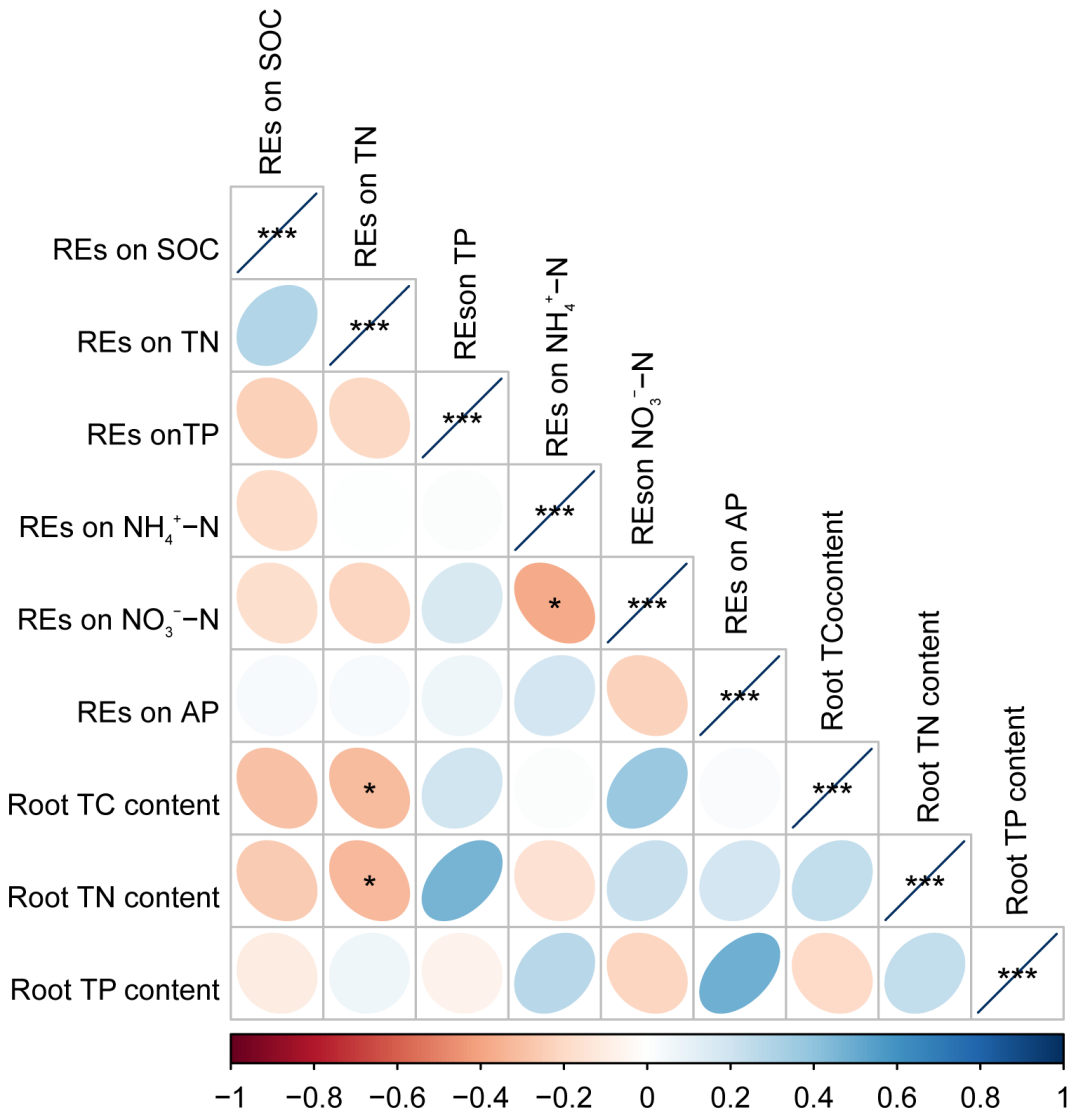
The matrix of Pearson’s correlation coefficients among paired REs and the chemical property of fine roots in *Pinus tabuliformis* forests. *p < 0.05, ***p < 0.001. REs, rhizosphere effects.

Pearson’s correlation analysis revealed that the root TC and TN contents were negatively correlated with the REs of SOC or REs of TN, respectively, but positively correlated with the REs of NO_3_^−^–N. However, these relationships were only significant in the REs of TN with root TC and TN contents (*p* < 0.05) (**Figure 8**). Root TN content also tended to decrease with the REs of NH_4_^+^–N; however, these relationships were not significant (*p* > 0.05) (**Figure 8**). In addition, the REs of NO_3_^−^–N were negatively correlated with the REs of NH_4_^+^–N (*p* < 0.05) (**Figure 8**).

### 3.4 Potential drivers of root N limitations during *P. tabuliformis* secondary succession

**Figure 9:**
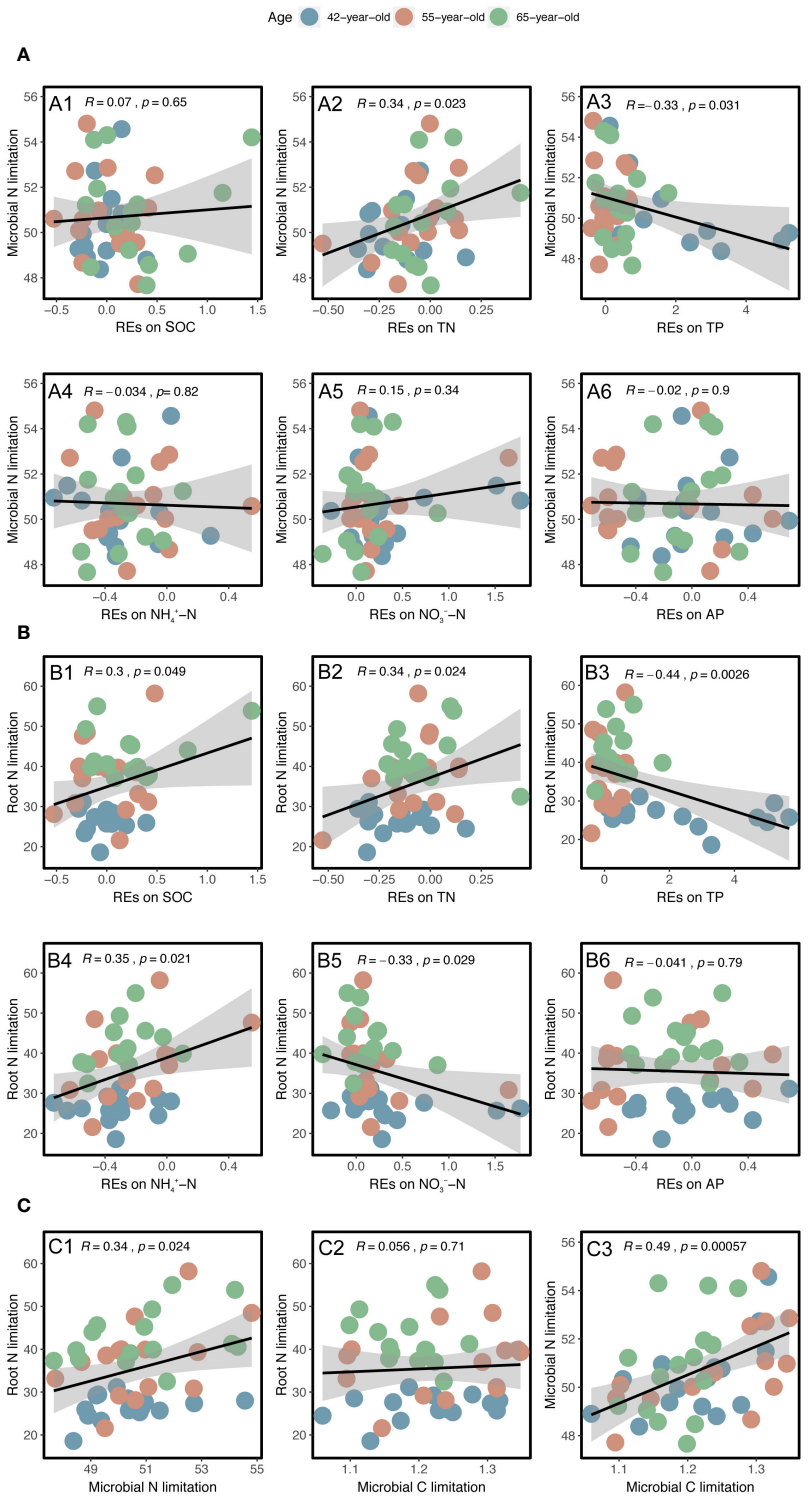
Linear regression analysis between the N limitations of rhizospheric soil microbes and root with the REs of each soil nutrient (SOC, TN, TP, NH_4_^+^–N, NO_3_^−^–N, and AP) **(A, B)**. Linear regression analysis between the N limitations of root and rhizospheric soil microbes and their relationships with the rhizospheric soil microbial C limitation **(C)**. The black lines with gray-shaded areas imply the least-squares linear regressions with the REs and limitations and their 95% confidence intervals. The values of R and *p* represent the corresponding Pearson’s correlation coefficients and level of significance, respectively. REs, rhizosphere effects; SOC, soil organic carbon; TN, total N; TP, total P; AP, available P.

**Figure 10:**
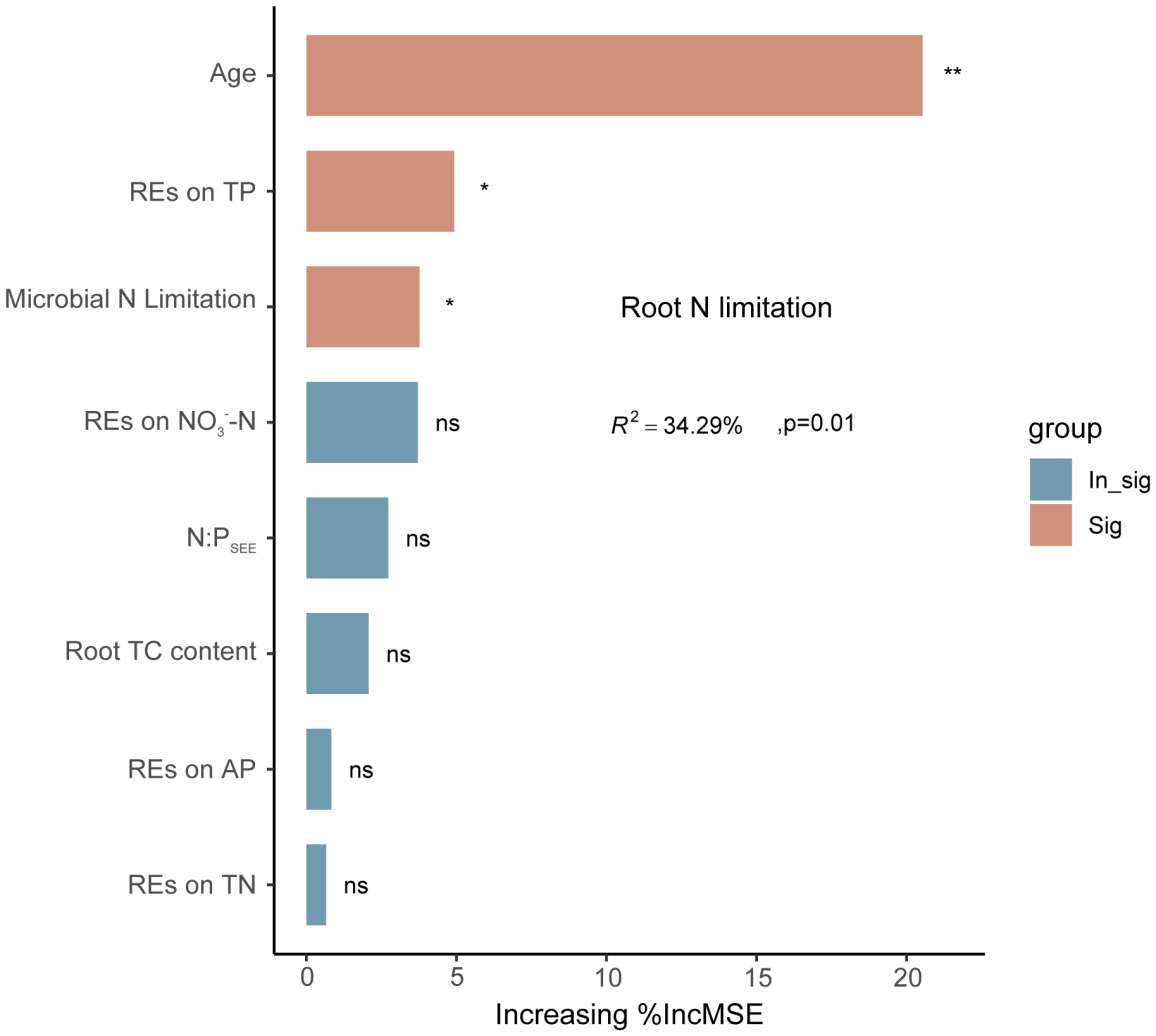
Potential drivers of variation in regulation of root N limitation in *Pinus tabuliformis* forests. Percentage increases in the mean square error (%MSE) were used to estimate the relative importance of these different predicted variables as drivers in regulation of root N limitation, and higher %MSE values mean more important predictors. *p < 0.05, **p < 0.01, ns (0.05 < *p* < 0.1).

Linear regression analysis indicated that REs of SOC, TN, TP, NH_4_^+^–N, and NO_3_^−^–N, as well as rhizospheric soil microbial N limitation, were linearly correlated with root N limitation, and their correlation coefficients were 0.30, 0.34, −0.44, 0.35, −0.33, and 0.34, respectively; *p* < 0.05) (**Figures 9B, C**). Among the variables, successional age (20.5% IncMSE), REs of TP (4.9% IncMSE), and microbial N limitation in the rhizosphere (3.8% IncMSE) were the three strongest predictors of root N limitation (**Figure 10**).

**Figure 11:**
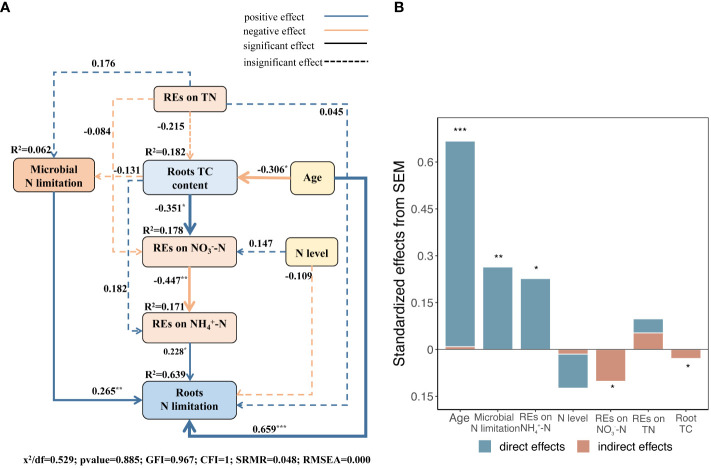
**(A)** Structural equation models (SEMs) describing multivariable effects of different predicted variables on REs of three different soil N-related indicators, i.e., total N, NH_4_^+^–N, and NO_3_^−^–N, limitations of rhizospheric soil microbes, successional ages, and roots TC content on root N limitation. Standardized path coefficients were expressed by numbers on the arrows. Proportion to the strengths of relationships is expressed by arrow widths. R^2^ represents the percentage of variance accounted for each response variable. CFI, GFI, and RMSEA represent comparative fit index, Jöreskog’s goodness fit index, and root mean square error of approximation, respectively. **(B)** Standardized direct, indirect, and total effects of each predicted variable on root N limitation calculated by SEM are demonstrated. *p < 0.05, **p < 0.01, ***p < 0.001.

The results of the SEM model demonstrated that the explanatory factors cumulatively explained 64 variances in root N limitation (**Figure 11A**). Succession, which had the highest overall effect, was the most critical factor driving root N limitation, both directly and indirectly. The N limitation of microbes in the rhizosphere and REs of NH_4_^+^–N availability, with relatively high total effects, ranked following succession and had direct positive effects on root N limitation (**Figures 11A, B**). Succession had an indirect effect on root N limitation by manipulating root TC content to regulate the REs of NO_3_^−^–N availability and the N limitation of microbes (**Figure 11A**). In addition, we found that the REs of TN directly and positively regulated microbial N limitation in the rhizosphere (**Figures 9A**, **11A**). However, the REs of TN and N levels had slight indirect effects on root N limitation (**Figures 11A, B**).

## 4 Discussion

### 4.1 Effects of N availability on N limitations of roots and microbes in the rhizosphere with succession

Initially, we found that the roots were limited by soil N, which is consistent with our previous study on branches and leaves (Yan et al., 2023). This indicates that both the aboveground growth and underground growth were consistent. In the present study, root N limitation was significantly alleviated when N addition rates reached 20 kg N m^−2^ year^−1^ in the 42-year and 55-year stands. However, N application increased root N limitation in the 65-year stands (**Figure 2I**). As a fast-growing tree species prior to maturity, the increase in exogenous N in *P. tabuliformis* directly promoted the accumulation of root TN in the 42-year and 55-year stands (**Figure 2C**), which is consistent with previous studies (Kou et al., 2018; Liu et al., 2021; Geng et al., 2023). However, because the increased growth rate of *P. tabuliformis* caused by improved N availability exceeds the nutrient uptake rates during the maturation period, dilution effects have been observed (Deng et al., 2017; Geng et al., 2023).

Notably, the microbial vector angles were (< 45°), indicating N restriction rather than P restriction for microorganisms in the rhizospheres of stands at the three successional ages (**Figures 3C, D**). Similarly, application of N generally alleviated microbial N limitations in the 42-year and 55-year stands but exacerbated them in the 65-year stand, although they were not significantly affected by the application of N due to their strong homeostasis (**Figures 3C, E, G**) (Zhang et al., 2019). This correlated with root N limitation and was consistent with the SEM results, which suggested a key role of microorganisms in plant nutrient limitation and competitive utilization of resources between roots and rhizospheric microbes (**Figure 11A**). Roots and microbes in the rhizosphere are always in progressive N limitation (**Figures 2I, J**, **3C, D**), which is attributed to the formation of strong depletion zones around the roots by continuous N uptake by plants (Kuzyakov and Xu, 2013). Similar patterns were also observed for root and microbial N limitation during succession, which increased with succession, consistent with previous studies (Zhou et al., 2017; Zhang et al., 2022), indicating that succession would increase N limitation in the root–soil–microbe system in the rhizosphere under the same level of soil N in this forest ecosystem.

### 4.2 Effects of N availability on the REs with succession

**Figure 12:**
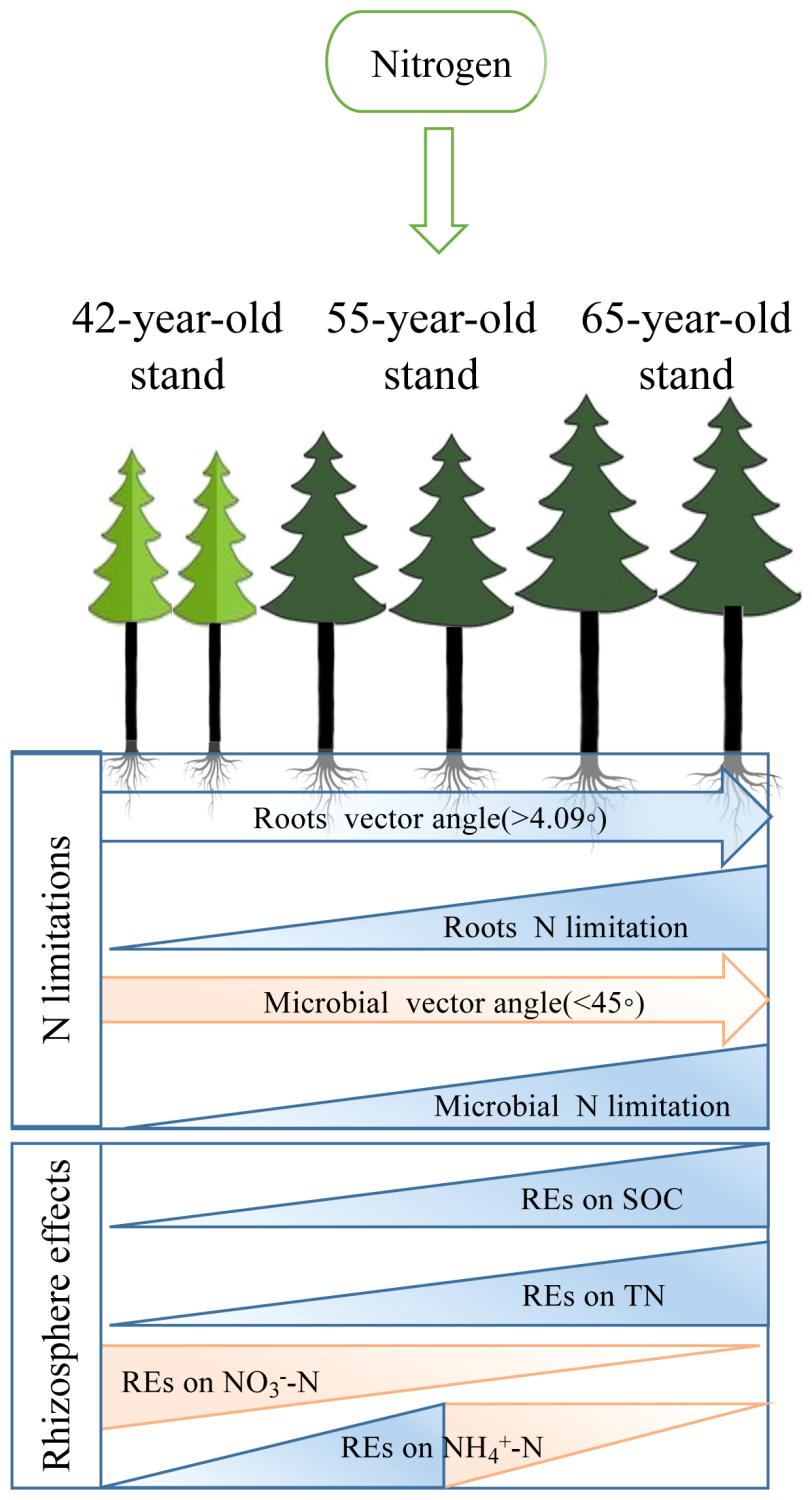
Conceptual framework for exploration of the pathways of successional effects on N limitations of roots and rhizospheric soil microbes after the application of N. We displayed linkages with REs of three different soil N-related indicators, i.e., total N, NH_4_^+^–N, and NO_3_^−^–N, to demonstrate that development with succession increased the N limitations of microbes and roots. REs, rhizosphere effects.

The REs of SOC and TN decreased following N application, although sensitivity to N varied in *P. tabuliformis* stands of different ages (**Figures 6A, C**). Soil, a strong source of plant N acquisition, provides N resources to meet its high demand and conserves N for low demand and *vice versa* (He et al., 2021). The application of N reduces the mineralization of soil N in the rhizosphere, which is aligned with C inputs from root exudates (Dijkstra et al., 2013; Cheng et al., 2014; He et al., 2021). Thus, the improvement in soil N availability resulting from exogenous N input (i.e., fertilization or atmospheric N deposition) would enable plants to obtain N from the soil immediately rather than investing more C in belowground tissues and root exudates to stimulate microorganisms to decompose SOM for N acquisition (Phillips et al., 2009). However, the magnitude of the REs of SOC and TN in the 65-year stand was the greatest (**Figures 6B, D**, **12**) in response to exacerbated root and microbial N limitations caused by lower root TC and TN contents in the roots of 65-year stands, in contrast to that in the 42-year and 55-year stands (Zhu et al., 2022) (**Figure 8**). This was consistent with some previous studies that showed that a dilution effect related to greater nutrient accumulation and biomass production would lead to lower N and P concentrations in plants (Guiz et al., 2016; Lü et al., 2019; Zhang et al., 2022). In addition to root exudates or rhizodeposits, microbes obtain N by assimilating the intractable decomposition of SOM to meet their elevated N requirements under intense N-limited conditions (Dijkstra et al., 2013; Kirkby et al., 2014; He et al., 2021), which is consistent with the preferential substrate utilization hypothesis (Phillips and Fahey, 2008; Dan et al., 2023).

The decreased REs of NH_4_^+^–N availability with the development of *P. tabuliformis* forests after the application of N was due to decreased root TN content (**Figure 8**), which is similar to previous findings (Hogberg et al., 1998; Jiang et al., 2020). However, positive REs of the NH_4_^+^–N availability in the 65-year stand and REs of the NO_3_^−^–N availability of all stands (**Figures 6G–G**, **7D, E**) positively direct root TC content to the REs of the NO_3_^−^–N, positively correlate between the REs of the NO_3_^−^–N and root TN content, and positively affect the indirect REs of the NO_3_^−^–N to NH_4_^+^–N ratio induced by grave root N limitation (**Figures 8**, **11A**) and demonstrated that differently aged natural secondary *P. tabuliformis* stands exhibited similar N preferences and dominantly relied on NO_3_^−^–N for its inorganic N nutrition. This was due to the greater mobile properties of NO_3_^−^–N than NH_4_^+^–N in soil solution (Kuzyakov and Xu, 2013). Our results also suggested that *P. tabuliformis* forests in 65-year-old absorbed NH_4_^+^–N from soils under severely N-limited conditions as a strategy to make full use of soil N sources, which enhances the primary productivity of forest ecosystems (Houle et al., 2014; Gao et al., 2020). Similarly, from this perspective, the REs of TN and NH_4_^+^–N availability alleviated root N limitation.

The REs of TP increased linearly with the N level, leading to a generally insignificant response signal of the REs of AP, which was further free from P-limited roots and microbes in the rhizosphere (**Figures 6K**, **7C**). Lower soil pH owing to the application of N promoted phosphatase activity, which is consistent with the findings of Phillips and Fahey (Phillips and Fahey, 2006).

### 4.3 REs of soil N components and rhizospheric soil microbial N limitations are useful predictors for root N limitations

**Figure 13:**
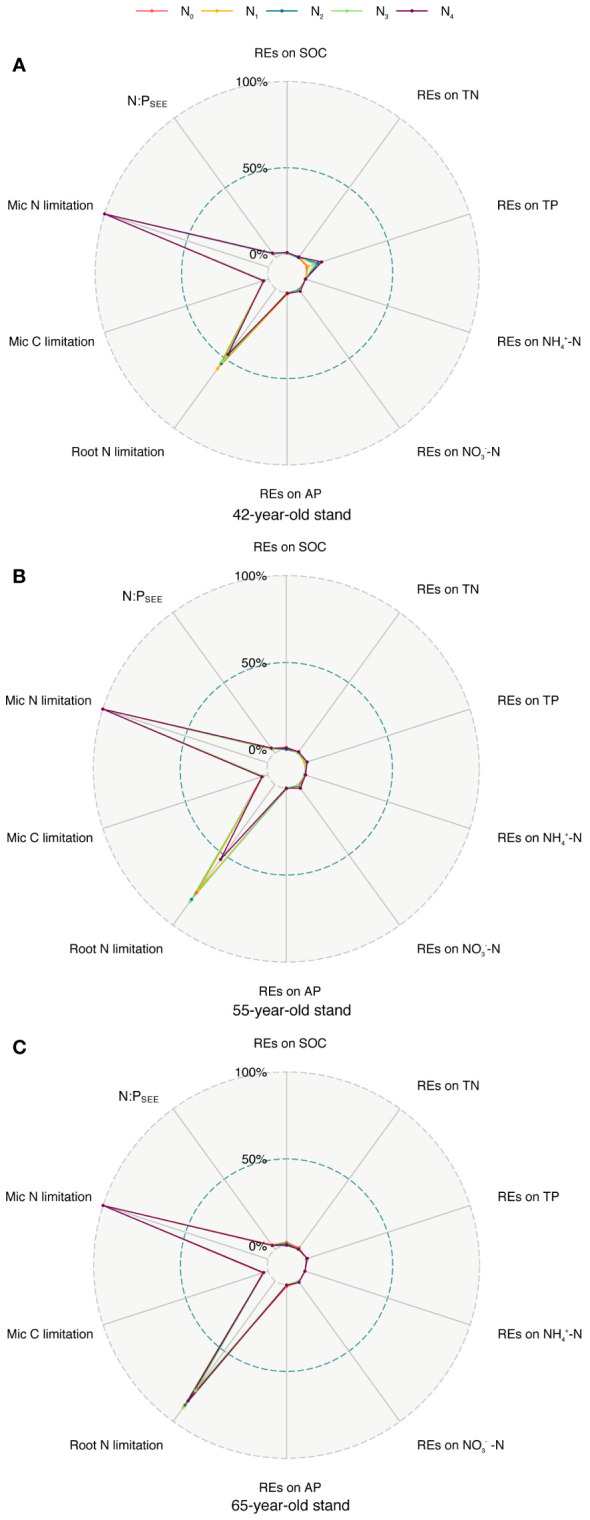
Comparison of REs of each soil nutrient (SOC, TN, TP, NH_4_^+^–N, NO_3_^−^–N, and AP), microbial C, N limitation, N:P_SEE_, and root N limitations among five N application levels of stands of 42-, 55- **(A)**, **(B)** and 65-year-old **(C)**. REs, rhizosphere effects; SOC, soil organic carbon; TN, total N; TP, total P; AP, available P.

As shown by root exudation and nutrient absorption, REs control N cycling in the soil. The REs of the soil N indicators were susceptible to soil N in *P. tabuliformis* stands at the three successional ages. Numerous studies have shown that applying N to soil has variable effects on each RE indicator of N indicators (Cai et al., 2023; Dan et al., 2023; Gao et al., 2023). Moreover, REs affect the nutrient supply to plants and are essential drivers for alleviating root N limitation (Sun et al., 2014; Han et al., 2023). Linear regression and RF analyses revealed the importance of the REs of soil N indicators and the N limitation of microbes in the rhizosphere for root N limitation (**Figures 9B, C**, **10**). SEMs revealed that the root TC content indirectly mitigated root N limitations by regulating the REs of NO_3_^−^–N availability and N limitations of microbes in the rhizosphere (**Figure 11A**). Thus, we put forward a conceptual diagram to summarize the effects of succession on the N limitation of roots and rhizospheric soil microbes (**Figure 12**). We determined the characteristics of stands of three successional ages by comparing their REs, microbial C and N limitations, and root N limitation among the five levels of N application using radar charts in *P. tabuliformis* forests after N input (**Figure 13**).

Stand development enhanced the fine root biomass production, resulted in weaker fine root nutrient foraging ability, and caused lower soil N nutrient availability (Ryan et al., 2004; Lü et al., 2019; Zhu et al., 2022). These changes directly decreased root TC and TN concentrations, increased investment in belowground photosynthetic products such as root exudates, and increased microbial N limitation in the rhizosphere symbiosed with roots when plant growth was N-constrained (**Figures 2B, D**, **3F, H**) (Guiz et al., 2016; Geng et al., 2021; He et al., 2021; Zhang et al., 2022; Geng et al., 2023). However, the increase in microbial N limitation with succession is insignificant owing to their faster turnover rates and strong homeostasis (Zhang et al., 2019, 2024). Root exudate inputs induced by succession promote the growth and activity of microorganisms and further stimulate stronger REs of SOC and TN and theoretically stronger REs of NH_4_^+^–N and NO_3_^−^–N availability to obtain more N resources (**Figures 6B, D**) (He et al., 2021; Zhu et al., 2022). In fact, continuous available N uptake by roots and microbes in the rhizosphere and the increased competitive utilization of resources between them led to strong depletion zones near the roots (Kuzyakov and Xu, 2013; Zhang et al., 2024), which ultimately decreased the REs of NH_4_^+^–N and NO_3_^−^–N availability with succession (**Figures 6H, J**). In addition, REs of NH_4_^+^–N also directly and positively drove the root N limitation and directly controlled the transformation from REs of NO_3_^−^–N (**Figure 11A**). This was because of the poor mobile properties in the soil solution of NH_4_^+^–N compared with NO_3_^−^–N and was not quickly absorbed by microbes in the rhizosphere but could be easily uptake by roots, making the roots outcompete in the uptake of N in the rhizosphere under N-limited condition (Kuzyakov and Xu, 2013; Huygens et al., 2016; Zhang et al., 2024).

The results of the radar charts showed that root N limitation of stands of the three ages and REs of TP in the 42-year stand generally increased with N levels, whereas root N limitation and REs of SOC greatly increased with succession (**Figure 13**). This result indicates that the adverse effect of N application on root N limitation in the 42-year stand was the smallest among the three stands of different ages. Therefore, the afforestation activities of the natural secondary *P. tabuliformis* forest in this area should prioritize middle-aged forests (40–50 years old) to adapt to global N deposition or the distribution heterogeneity of bioavailable soil N.

## 5 Conclusion

We assessed the responses of N limitation in the roots and rhizospheric soil microbes and the REs to N application of N-limited natural secondary *P. tabuliformis* forests of different successional ages. N limitation characterized the root and microbial communities. Older tree stands were subject to relatively strong root and microbial N limitations compared to younger stands in response to N. The magnitudes of the REs of SOC and TN were significantly greater in the 65-year stands. However, the magnitudes of the REs of P indicators and available N indicators, which were NH_4_^+^–N and NO_3_–N, were significantly lower in the 65-year stand.

Root N limitation was primarily contingent on variations in the REs of three different soil N-related indicators, TN, NH_4_^+^–N, and NO_3_^−^–N. Rhizospheric soil microbial N limitation was one of the most important factors driving root N limitation. The level of N application had only a slight effect on root N limitation. In addition, the root TC content, driven by successional age, directly affected microbial N limitation. This suggested that succession of *P. tabuliformis* forest increased microbial metabolic N limitation and further increased root N limitation owing to the formation of strong depletion zones near the roots but stimulated stronger REs to obtain more N in a N-limited forest ecosystem.

## Data availability statement

The raw data supporting the conclusions of this article will be made available by the authors, without undue reservation.

## Author contributions

SD: Conceptualization, Formal analysis, Investigation, Software, Writing – original draft, Writing – review & editing. JG: Funding acquisition, Supervision, Writing – review & editing. YZ: Formal analysis, Investigation, Writing – review & editing. LL: Investigation, Writing – review & editing. RW: Investigation, Writing – review & editing. RZ: Investigation, Writing – review & editing.
